# Filshie clip migration with multiple groin hernias: a case report

**DOI:** 10.1186/s13256-015-0665-x

**Published:** 2015-09-07

**Authors:** Alison Michelle Mumme, Jamie Cham

**Affiliations:** 1 Department of Surgery, Wagga Wagga Base Hospital, Wagga Wagga, NSW 2650 Australia; 2 The University of New South Wales, Rural Clinical School, Wagga Wagga, NSW Australia

**Keywords:** Contraception, General surgery, Hernia, Tubal occlusion

## Abstract

**Introduction:**

Tubal occlusion is a common form of contraception. Filshie clips have been widely used for tubal occlusion since their introduction. Reports of Filshie clip migration are rare. We describe what we believe to be the first reported case of a patient with multiple groin hernias associated with migration of a Filshie clip.

**Case presentation:**

We report the case of 56-year-old Caucasian woman who presented with a tender right groin lump. She had undergone a right-sided inguinal hernia repair 3 years earlier. Tubal occlusion had been performed using Filshie clips 21 years prior. Computed tomography revealed a tubal clip within her right inguinal region, and had also been identified on imaging prior to a previous hernia repair. Our patient underwent repair of a right femoral hernia, with the tubal clip identified in the sac and removed. She has since had no recurrences.

**Conclusion:**

Filshie clip migration is a rare event, with occasional complications occurring. This case highlights the importance of identification and removal of such foreign bodies, potentially reducing the risk of hernia recurrence or further complications.

## Introduction

Since the introduction of the Filshie clip in the 1980s, they have been widely used for tubal occlusion in female sterilization [1]. Tubal occlusion is the preferred method of 9.7% of Australian women using contraception [2]. Case reports of Filshie clip migration show the timeframe between initial sterilisation and development of complications varies between 6 weeks and 15 years [3, 4].

Hernia repair is a commonly performed surgical procedure. Groin hernias are the most prevalent with over 40,000 Australians undergoing groin hernia repairs each year [5]. Hernias caused by foreign bodies are an unusual but reported event [6, 7].

The pathophysiological process by which foreign bodies cause hernias is still not understood, with multiple hypotheses suggested [4, 6, 8]. We describe multiple groin hernias postulated to have developed in a patient secondary to migration of a Filshie clip placed during tubal occlusion 21 years previously.

## Case presentation

A 56-year-old Caucasian woman presented to our emergency department with a tender right groin lump. Previous surgical history included a right-sided open inguinal hernia repair using mesh 3 years prior, which was complicated by chronic right inguinal seroma. She had undergone drainage of the seroma on one occasion, with other recurrences resolving without intervention. This hospital admission was owing to increased pain compared to her “usual” seroma pain, as well as her inability to reduce the lump. Tubal occlusion using Filshie clips had been performed 21 years prior. There was no other relevant medical history. On examination she had normal hemodynamics. Her abdomen was soft and not distended. She had a tender lump in her lower groin that was irreducible had no cough impulse. There were no overlying skin changes.

**Fig. 1:**
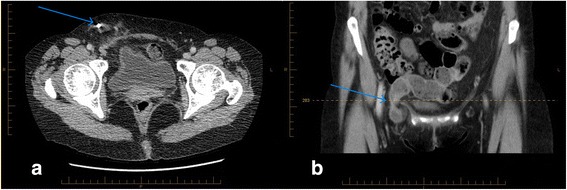
Computed tomography imaging of the abdomen and pelvis showing the tubal clip (blue arrow in **a**) and small bowel extending out of the pelvis (blue arrow in **b**)

Computed tomography revealed a small seroma, with a tubal clip within her right inguinal region, in subcutaneous tissues (Fig. 1). A left tubal clip remained in place. This was consistent with imaging from previous admissions. Additionally in this region our patient had small bowel extending out of her pelvis. While awaiting surgery, our patient experienced increased pain, vomiting, nausea, and anorexia.

Our patient underwent repair of a right groin hernia. A femoral hernia was identified and strangulated omentum excised. A closed metal tubal clip was located in the hernial sac and removed. This clip proved elusive during the initial hernia operation. The defect was closed using mesh repair. Our patient had an uneventful postoperative course and was discharged on day 1.

Hysterosalpingography was considered to check tubal patency but not performed.

## Discussion

Filshie clip migration is a rare complication of tubal occlusion, with the incidence reported between 0.1 and 0.6% [9]. Wong *et al*. reported Filshie clips within hernias, external to the hernial sac. To the best of our knowledge, there is only one previous case study reporting a Filshie clip within a hernial sac [6].

The Filshie clip sterilizes woman by clamping the fallopian tubes, causing avascular necrosis on both sides of the tube. This separates the tube into two stumps, with the Filshie clip usually remaining attached, covered by peritoneum. Occasionally clips can become mobile and migrate to other areas of the abdomen, predominately the para-colic gutters or the recto-uterine pouch [10]. In other cases, they can cause abscesses, fistulae, urinary tract disturbances, and, in rare cases, hernias [9]. The mechanism by which surgical clip migration takes place remains unclear but it may be affected by technical factors associated with clip placement as well as the number of clips inserted [11].

An abdominal hernia may be complicated by the presence of a foreign body in the hernia sac or adjacent to the hernia mass. A proposed mechanism of foreign body migration is via initiation of an inflammatory reaction, which may have two effects: weakening the parietal peritoneum, predisposing to hernias; and creating a mass of granulomatous, fibrous tissue that may herniate [12]. The source of the foreign body may be from the intestinal lumen, via penetration of the abdominal wall, or as a consequence of surgery, whether purposefully with surgical clips or unintentionally with sponges or instruments left in the body.

There is some controversy as to whether Filshie clips cause hernias, or whether they are simply found in hernias. Garner *et al*. [6] proposed that Filshie clips are likely to migrate to the site of the femoral hernia due to gravity; however, the presence of the clips in ventral abdominal wall hernias does not support this hypothesis of clip migration [4]. In our case, the development of two groin hernias, in different locations containing the Filshie clip, suggests that the clip played a role in the etiology of the hernia. One year after Filshie clip removal and hernia repair, our patient has been asymptomatic with no further hernia formation.

## Conclusion

Filshie clip migration is a rare event with several complications, including the possibility of hernia formation. When foreign bodies are located in a hernial sac, identification and removal of the foreign body should occur in addition to repair of the hernia. Doing so potentially reduces the risk of hernia formation, recurrence, or additional complications secondary to foreign body migration.

## Consent

Written informed consent was obtained from the patient for publication of this case report and accompanying images. A copy of the written consent is available for review by the Editor-in-Chief of this journal.
